# Impact on Dietary Intake of Two Levels of Technology-Assisted Personalized Nutrition: A Randomized Trial

**DOI:** 10.3390/nu12113334

**Published:** 2020-10-29

**Authors:** Megan E. Rollo, Rebecca L. Haslam, Clare E. Collins

**Affiliations:** 1 Priority Research Centre for Physical Activity and Nutrition, The University of Newcastle, Callaghan, NSW 2308, Australia; rebecca.williams@newcastle.edu.au; 2 School of Health Sciences, Faculty of Health and Medicine, The University of Newcastle, Callaghan, NSW 2308, Australia

**Keywords:** behavioral nutrition intervention, digital health, personalized nutrition, telehealth

## Abstract

Advances in web and mobile technologies have created efficiencies relating to collection, analysis and interpretation of dietary intake data. This study compared the impact of two levels of nutrition support: (1) low personalization, comprising a web-based personalized nutrition feedback report generated using the Australian Eating Survey^®^ (AES) food frequency questionnaire data; and (2) high personalization, involving structured video calls with a dietitian using the AES report plus dietary self-monitoring with text message feedback. Intake was measured at baseline and 12 weeks using the AES and diet quality using the Australian Recommended Food Score (ARFS). Fifty participants (aged 39.2 ± 12.5 years; Body Mass Index 26.4 ± 6.0 kg/m^2^; 86.0% female) completed baseline measures. Significant (*p* < 0.05) between-group differences in dietary changes favored the high personalization group for total ARFS (5.6 points (95% CI 1.3 to 10.0)) and ARFS sub-scales of meat (0.9 points (0.4 to 1.6)), vegetarian alternatives (0.8 points (0.1 to 1.4)), and dairy (1.3 points (0.3 to 2.3)). Additional significant changes in favor of the high personalization group occurred for proportion of energy intake derived from energy-dense, nutrient-poor foods (−7.2% (−13.8% to −0.5%)) and takeaway foods sub-group (−3.4% (−6.5% to 0.3%). Significant within-group changes were observed for 12 dietary variables in the high personalization group vs. one variable for low personalization. A higher level of personalized support combining the AES report with one-on-one dietitian video calls and dietary self-monitoring resulted in greater dietary change compared to the AES report alone. These findings suggest nutrition-related web and mobile technologies in combination with personalized dietitian delivered advice have a greater impact compared to when used alone.

## 1. Introduction

Sub-optimal dietary intake has a well-established relationship with increased risk of non-communicable diseases and mortality [1,2,3]. In 2017, dietary risks accounted for 10.9 million (95% uncertainty interval 10·1–11·7) deaths and 255 million (234–274) disability-adjusted life-years, and were the leading cause of mortality and second leading cause of mortality among level two risk factors, respectively [4]. Diet-related burden of disease was highest for cardiovascular diseases, followed by cancers and type 2 diabetes [5]. Further, modelling of Australian data estimates that by 2025, inadequate fruit and vegetable intakes could result in total productivity losses of AUD 498 million [6].

Technology-assisted tailoring and delivery of feedback and advice in relation to diet, and to promote healthy eating, has been shown to be effective [7]. Recently the term “personalized nutrition” has emerged as an extension of tailored nutrition advice, which is often personalized based on nutritional requirements for age, sex and life stage (i.e., pregnant or breastfeeding), nutritional status (e.g., Body Mass Index (BMI)), and the presence of chronic disease (e.g., reduced sodium intake in hypertension). Personalized nutrition is where more individual level characteristics are considered when providing dietary advice. Genomics has expanded the scope of personalized nutrition with dietary prescription based on genotype, in addition to phenotype and adequacy of intake [8]. The Food4Me project based in seven European countries aimed to capitalise on improved access to the internet to evaluate an online personalized dietary intervention [9]. Personalization of dietary advice delivered via the internet was more effective than generic population healthy eating information; however, adding phenotypic or genotypic data did not provide any additional benefit to personalizing interventions based on dietary intake alone [10]. At this time, the impact of genotype-based personalized nutrition to motivate or engage individuals to change dietary intake remains unclear [11]. 

Foods and nutrients consumed are the outcome of the individual’s eating behavior, which in turn is influenced by multiple internal and external factors. Exploration of new variables to extend personalized nutrition are needed, in particular those focused on the individual behavioral level. The primary aim of this intervention trial was to compare the impact on dietary intake of two technology-assisted nutrition interventions with differing levels of personalization. A secondary aim was to evaluate participant’s perceptions of acceptability of intervention components. 

## 2. Materials and Methods

### 2.1. Study Design

This study was a pilot randomized trial with participants randomized into two intervention groups: (1) low personalization, or (2) high personalization. Ethical approval was received from the University of Newcastle Human Research Ethics Committee (H-2015-0217) with participants providing written informed consent. 

### 2.2. Participants and Recruitment

Participants were recruited via a combination of print (e.g., flyers around the university campus) and online materials (e.g., media release, social media), which invited interested individuals to test a new web-based dietary assessment tool providing personalized feedback on food and nutrient intakes. To be eligible to participate, individuals needed to be aged ≥18 years; not pregnant, breastfeeding or trying to conceive; weight stable (±4 kg) in past 3 months; and have no dietary restrictions or medical conditions requiring intensive medical nutrition therapy. In addition, participants needed to have broadband internet access (minimum connection speed tested), equipment for participating in video calls, and have access to a smartphone that regularly connected to a WiFi network. 

### 2.3. Intervention Groups

#### 2.3.1. Low Personalization Group

Participants allocated to the low personalization (LoP) group received a personalized dietary intake feedback report based on their responses to the Australian Eating Survey^®^ (AES) food frequency questionnaire (FFQ) [12]. Further details on the use of the AES to assess dietary intake are provided in Section 2.4.1. The AES FFQ transitioned to a web-based application, enabling response data to be automatically analysed and a personalized dietary intake feedback report generated immediately following completion. 

**Figure 1:**
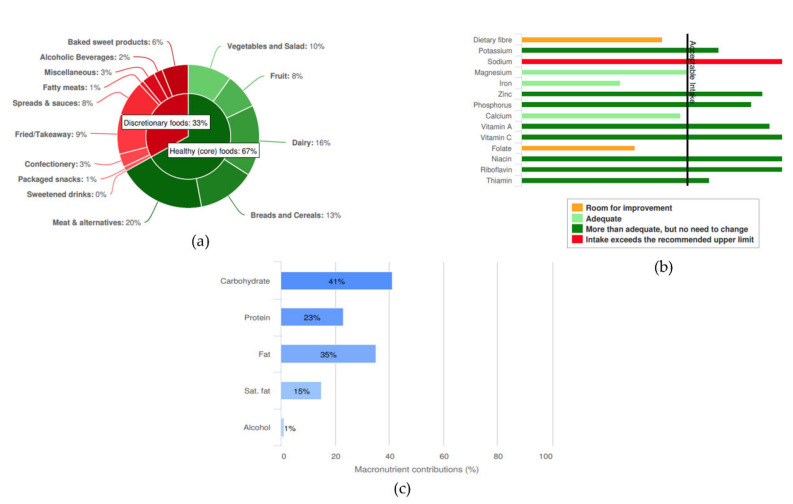
Examples of graphical feedback provided in the Australian Eating Survey^®^ (AES) report: (**a**) proportion of energy intake from core and non-core food groups; (**b**) adequacy of fibre and micronutrient intake; and (**c**) proportion of energy intake from macronutrients.

The AES feedback report compares the individual’s usual dietary intake to Australian food and nutrient recommendations, summarizing the adequacy of intake through a combination of text, tables and graphs. The first section of the report summarizes food intake in the form of the contribution of core (healthful, nutrient-dense) and non-core (energy-dense, nutrient-poor) foods as a percentage of total energy intake (%EI), and also subgroup level (e.g., %EI from core foods is comprised of %EI from fruits, vegetables, dairy, grains, meat and meat alternatives). In addition, feedback on variety within core food groups is provided by the Australian Recommended Food Score (ARFS) [13] (see Section 2.4.1). The second section of the report compares macro- and micronutrient intakes to Nutrient Reference Values (NRVs) [14]. The proportion of total energy intake of carbohydrate, protein, fat, saturated fat and alcohol is presented graphically along with the Acceptable Macronutrient Distribution Ranges. Intakes of dietary fibre and micronutrients are presented as both absolute values (e.g., mg/day) and in graphical form with absolute intake for each respective nutrient compared to the nutrient’s NRV for the individual’s age, gender, and for females, pregnancy and breastfeeding status. Adequacy of intake is indicated via a diagram where the color orange = “room for improvement”; light green = “adequate”; dark green = “more than adequate, but no need to change”; red = “Intake exceeds the recommended upper limit”. The final section of the report provides a list of key food sources for dietary fibre and 13 micronutrients to assist users with food choices to help optimise nutrient intakes. Figure 1 provides examples of the graphs included in the feedback report, summarizing food and nutrient intake in comparison to recommendations. 

Participants in the LoP group received access to their AES feedback report immediately following completion of the AES FFQ. Participants were also sent a copy of their report via email from a member of the research team. The email instructed participants to use the AES feedback report to self-identify areas in their diet they would like to change and make any changes that they saw fit to address this area. In week six, LoP participants were asked to complete another AES FFQ, receiving the dietary feedback report following survey completion and asked to use the report to guide any further dietary changes. 

#### 2.3.2. High Personalization Group

High personalization (HiP) participants also completed the AES FFQ at baseline. However, their AES dietary feedback report was not available immediately. Rather, participants were guided through their feedback report during a video call with a dietitian in week one. Prior to this call, participants were emailed and asked to complete a personalized nutrition questionnaire (PNQ), a tool to support the dietitian to deliver the video coaching by identifying and prioritising factors that the individual perceives as affecting their ability to eat healthy. The PNQ is based on the behavior change wheel, which outlines the interrelationships between capability (C), opportunity (O) and motivation (M) and its impact on behavior (B) [15]. The generic COM-B questionnaire presented by Michie, Atkins and West ([16]) was used as the framework for development of the PNQ, with included factors relating to barriers associated with healthy eating (e.g., increased vegetables and fruit, decreased junk foods). The PNQ is comprised of 18 factors across physical and psychological capacity (7 factors), environmental and social opportunity (5 factors), and reflective and automatic motivation processes (6 factors). After selecting relevant PNQ factors, participants ranked the top three factors they wished to prioritise. An accompanying toolbox resource mapped each item of the PNQ to the intervention functions and associated behavior change techniques of the CALO-RE taxonomy [17], and included sample intervention strategies that the dietitian could use to support behavior change elements. 

Participants were allocated up to four video coaching calls with a dietitian scheduled for weeks one, three, six and nine. The initial video call with the dietitian in week one was a structured 30 min consultation. Participant responses to the PNQ, along with data collected during the baseline measurement session (e.g., height, weight), motives for participation, and the AES feedback report were provided to the dietitian for review ahead of the first consultation. The dietitian used these data to initiate a brief discussion on the participant’s PNQ responses and results from the AES report in order to set goals. The goals included one long-term goal (12 week timeframe; nutrition-related outcome or dietary behavior focused) and three short-term goals (~2 week timeframe; focused on behaviors impacting intake). All goals were personalized to the participant, however one short-term goal was pre-determined and aimed to optimise servings and variety of vegetables and fruits, and limiting energy-dense, nutrient-poor (non-core) food choices in accordance with Australian dietary guidelines [18]. Following the setting of goals with the participant, the dietitian used the PNQ responses and accompanying toolbox to select appropriate strategies in consultation with the participant and formed an action plan. Perceived challenges to implementation of the plan were identified and possible solutions explored. The session ended with the dietitian summarizing participant goals and action plans, with the confirmed information and AES feedback report sent to the participant via email on completion of the video coaching session. 

In week six, participants were required to complete another 30 min video call with the dietitian, which followed a structure similar to week one. Two optional video coaching sessions were offered to participants in weeks three and nine. These sessions were shorter in duration (up to 15 min each) and provided an opportunity for participants to “check-in” with the dietitian to receive additional support on barriers and problem solving, and adjustment of goals and strategies based on participant progress. 

The second intervention component in the HiP group was dietary self-monitoring and text message feedback. Participants self-monitored their dietary intake for a minimum of 3 days/week using an image-based food record methodology adapted from previous studies [19,20,21,22]. Participants used the Evernote^®^ app on their smartphone to capture images of food and drink items plus a text description of the items prior to consumption. Reminder text messages encouraging participants to self-monitor using the app were sent at the start of each week. Participant image-based food records were uploaded automatically and analysed using a dedicated tool by members of the research team trained in image-based record analysis. Feedback on the previous week’s intake was provided to participants weekly, except for weeks one, four and ten via three text messages related to servings of vegetables, fruit and energy-dense, nutrient-poor foods (non-core) in comparison to guidelines [18]. Standardised message templates were used based on a previous study that provided tailored text messages relative to intakes of selected food groups [23]. In weeks four and ten, participants received a text message asking to reflect on progress towards their short-term and long-term behavioral goals and use their image-based food record to assist in this process. 

### 2.4. Outcome Measures

#### 2.4.1. Dietary Intake Assessment

Participant food and nutrient intake was assessed pre- and post-intervention (at baseline and 3 months, respectively) using the AES, a 120 item semi-quantitative food frequency questionnaire that assesses usual dietary intake over the past 3–6 months [12]. The frequency options within the AES ranged from “Never” up to “≥4 times/day”, but varied depending on the food, with some drinks items up to “≥7 glasses/day”. 

Change in overall ARFS was the primary outcome for this study. The ARFS assesses diet variety within the core food groups and has been validated in healthy adults [13]. The ARFS uses a sub-set of 70 AES food items and comprises eight sub-scales from a range of healthful or core food groups (e.g., vegetables, fruit, grains, meats, non-meat proteins, dairy) with total score ranging from 0 to 73. For most items, AES frequency response options are collapsed into two categories “once per week or more” or “less than once per week or never”. A higher total score is indicative of greater variety within the core food groups and more optimal nutrient intakes. Secondary outcomes related to other changes in macronutrient and micronutrient intake, with nutrient intakes, were computed using the Australian Food, Supplement and Nutrient database (AUSNUT 1999) [24]. Standard adult portion sizes for each food item were derived from National Nutrition Survey data or from the product standard serving size (e.g., slice of bread) [25]. In addition, change in the distribution of total energy intake from foods categorized into two main groups core (e.g., fruits, vegetables, grains) and non-core (e.g., sweetened drinks, confectionary, takeaway foods) and their respective sub-groups was also evaluated.

#### 2.4.2. Feedback on Intervention Components

Following the 12 week intervention period, participants completed a process evaluation questionnaire on the relevant intervention components. Both groups answered questions on use and acceptability of the online AES and associated dietary feedback report, and whether any other resources in addition to those provided in this study were used to assist with making dietary changes. In addition, the HiP group were also asked questions on their experience of the dietitian video coaching sessions and use of the image-based food record for dietary self-monitoring and associated text message feedback received on vegetables, fruit, and energy-dense, nutrient-poor food intakes. The majority of questions asked participants to rank level of agreement on a 5-point Likert scale (“strongly disagree”, “disagree”, “neither disagree or agree”, “agree”, “strongly agree”). Other questions relating to impact and satisfaction with coaching calls, dietary self-monitoring and text message feedback were also asked.

#### 2.4.3. Other Measures

Participants attended two in-person sessions where physical measures, including height and weight, were obtained. Weight was measured in light clothing without shoes to 0.01 kg on a digital scale (Inbody 720; Biospace Co., Ltd., Seoul, Korea). Height was measured without shoes to 0.1 cm using the stretch stature method on a stadiometer (Inbody BSM370; Biospace Co., Ltd., Seoul, Korea). In addition, a baseline questionnaire collected general data (e.g., age, education level, household income, marital status) and experience with using the internet for health information via the eHealth Literacy Scale (eHEALS) [26]. 

### 2.5. Statistical Analysis

Data were analysed using IBM SPSS Statistics for Windows, version 24.0 (IBM Corporation, Armonk, NY, USA). Participant baseline characteristics and process evaluation descriptive statistics are reported as mean (SD) or as count (%). The effect of treatment on the primary (ARFS: overall score) and secondary outcomes were assessed using linear mixed models. The effects were time (baseline and 3 months), intervention group (HiP and LoP) and a group x time interaction term. A compound symmetry residual covariance structure was used model dependence between the repeated measurements within each subject. Age, gender and baseline BMI were added as covariates and, when significant, their interactions with time and group were examined. Significant covariates and any significant interactions remained in the final model for determination of adjusted between-group differences. Statistical significance was set at the 0.05 level. 

## 3. Results

### 3.1. Participant Baseline Characteristics and Dietary Intakes

**Figure 2:**
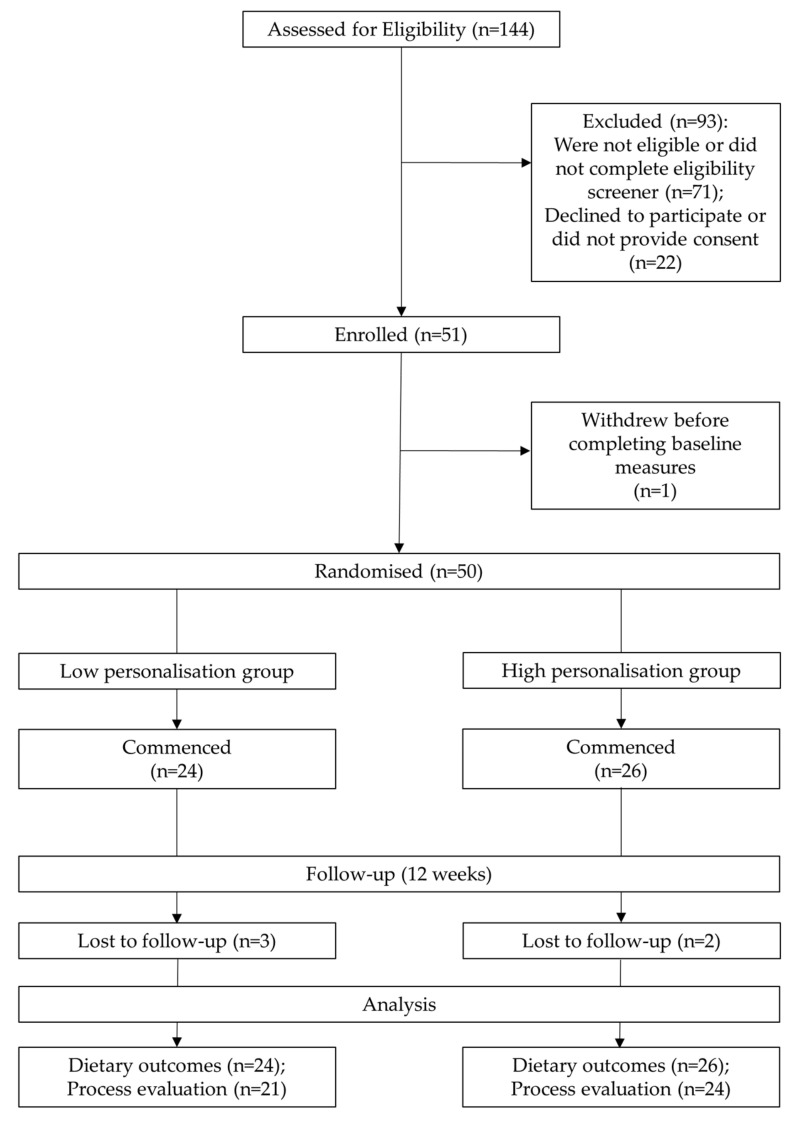
Flow diagram of study participants.

Fifty-one participants were enrolled into the study at baseline. Of these, one participant withdrew and did not complete the baseline dietary assessment component. Therefore, data are presented for participants with complete baseline measures (*n* = 50). Two participants from the HiP group and three from LoP group did not participate in the follow-up at the end of the 12 week intervention period. In addition, two HiP participants did not complete process evaluation questionnaires. Figure 2 summarizes the flow of participants through the study.

At baseline, participants were predominantly female (86.0%), mean (SD) age of 39.2 (12.5) years and with a BMI of 26.4 (6.0) kg/m^2^. Fifty percent had a postgraduate degree and were in full-time employment (42.0%). Overall, self-reported eHealth literacy scored moderately high at 29.8 (5.0) points out of 40 points. Table 1 summarizes baseline characteristics of participants by group. 

The dietary intakes of participants at baseline are presented in Table 2 and Supplementary Table S1. ARFS scores (overall and sub-scales) were similar between the two groups. The HiP group had a higher proportion of total energy intake from non-core (energy-dense, nutrient-poor) foods compared to the LoP group (35.3 ± 11.8% vs. 30.9 ± 13.5%, respectively), while the LoP group consumed a diet that had a higher proportion of energy intake as alcohol compared to the HiP group prior to the intervention (3.6 ± 5.7% vs. 2.3 ± 3.9%, respectively).

### 3.2. Dietary Intake Outcomes

Table 3 and Supplementary Table S2 summarize dietary intake at baseline for all participants and within- and between-group differences following the 12 week intervention period. Change in the primary outcome, total ARFS, was statistically significant at 12 weeks in favor of the HiP group (5.6 points (95% CI 1.3 to 10.0) and ARFS sub-scales of meat (0.9 points (0.4 to 1.6)), vegetarian alternatives (0.8 points (0.1 to 1.4)), and dairy (1.3 points (0.3 to 2.3)).

Additional significant changes in favor of the HiP group compared to LoP group occurred for percentage of total energy intake from all energy-dense, nutrient-poor foods (non-core foods) (−7.2% (−13.8% to −0.5%)), core foods 11.7% (7.2% to 16.2%) and takeaway foods sub-group (−3.4% (−6.5% to 0.3%)). Group × time interactions were statistically significant in favor of the HiP group for the same six variables. Significant within-group changes were observed for 12 dietary variables in the HiP group versus one variable for LoP.

### 3.3. Perceptions of the AES and Feedback Report

**Figure 3:**
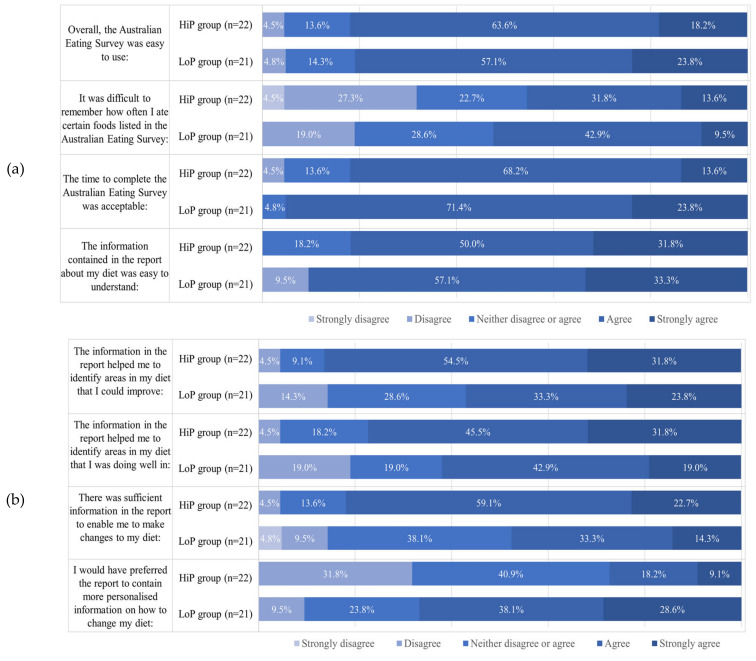
Participant feedback on the AES (**a**) and associated dietary feedback report (**b**).

Figure 3a,b summarizes participant responses after completing the AES FFQ and on usefulness of the personalized dietary intake feedback report. The majority of participants in both groups found the AES easy to use, completion time acceptable (>80% strongly agreed or agreed), and the information in the dietary feedback report easy to understand (Figure 3a). A higher proportion of participants in the LoP group strongly agreed or agreed with the statement that it was difficult to remember how often some foods were eaten compared to the HiP group (52.4% vs. 45.4%, respectively). Differences between groups existed for the usefulness of the AES report in assisting with changing intake with a higher proportion of participants in the HiP group indicating that they strongly agreed or agreed with these statements compared to those in the LoP group, for most questions (Figure 3b).

All participants were asked if they sought any additional information or assistance to make any dietary changes, either alone or in combination with resources provided as part of study participation. Seven participants (HiP *n* = 2 and LoP *n* = 5) sought additional advice from a health professional, with “dietitian” and “exercise physiologist” the most common type of health professional listed. Twenty participants (HiP *n* = 12 and LoP *n* = 8) sought additional healthy eating information predominantly from specific websites and/or via an internet search and 16 participants (HiP *n* = 6 and LoP *n* = 10) used additional smartphone apps predominantly to monitor intake. 

### 3.4. Participant Perceptions of Dietitian Video Coaching Sessions

All 26 participants in the HiP group completed the initial video call coaching session with the dietitian. Of these only 11 (36.7%) participants completed the optional week three session, while 20 (76.9%) participants completed the mid-point session at 6 weeks. No participants requested the final optional session in week nine. 

Perceptions on the impact of and satisfaction with the dietitian consultations are summarized in Supplementary Table S3 for the 22 HiP participants who completed the process evaluation. Most participants found the consultations useful for providing information, increasing confidence and assisting with achieving goals. Most participants either strongly agreed or agreed that goals were personalized, and strategies addressed personal barriers to healthy eating. 

Twenty participants felt the number of sessions were “just right”, while the remaining two participants indicated that they “would have preferred more regular contact”. All participants reported the duration of consultations was sufficient. Compared to interacting with a health professional in-person, participants reported being very comfortable (*n* = 9) or comfortable (*n* = 7) interacting online, while three were “neutral”. Overall, nine participants were “extremely satisfied”, seven “very satisfied” and five “moderately satisfied” with the video coaching sessions with the dietitian, while the remaining person was “slightly satisfied” (*n* = 1).

### 3.5. Perceptions of Dietary Intake Self-Monitoring and Text Message Feedback

Twenty-five HiP group participants used the Evernote^®^ app to collect image-based food records for self-monitoring dietary intake at least once during the intervention. Supplementary Tables S4 and S5 summarize responses (*n* = 22) from the process evaluation of the dietary self-monitoring and text message feedback received by the HiP group. The majority of participants reported that it was easy to capture an image; however, remembering to record food and drinks before eating and recording if any leftovers were present was difficult for most participants. Over three-quarters of participants felt using the image-based record to self-monitor increased their awareness of the types and amounts of foods eaten and the frequency of eating. Most participants (59.1%) reported that use of an image-based food record was an acceptable form of dietary self-monitoring.

Perceptions of HiP group participants of the weekly text messages consisting of dietary feedback on image-based food records and reminders relating to goals are reported in Supplementary Table S5. For a high proportion of participants (81.8% to 100.0%), the text message feedback on fruit and vegetable variety and amounts of energy-dense, nutrient-poor (“non-core”) food intake prompted participants to consider their intake of these foods. Half the participants felt the text messages assisted them in achieving their diet-related goals, with 59.1% indicating messages only had a short-term effect and 50.0% indicating text messages made them feel better about their diet.

When asked to rate overall on a scale of 1 to 10 (1 = “not at all motivated”, 10 = “extremely motivated”) how much the text messages motivated them to take action and change their diet, 15 participants rated the messages as “extremely motivated”, six participates rated their motivation as “5” or “2” (three participants each), and one participant rated their motivation as a “3”. Finally, when asked to report overall satisfaction with the text message feedback, two participants reported they were “extremely satisfied”, seven participants were “very satisfied”, eight were “moderately satisfied”, four were “slightly satisfied”, and one was “not at all satisfied”.

## 4. Discussion

This study evaluated the impact on dietary intake of two levels of personalization, delivered using web and mobile technologies. Higher level personalization consisting of one-on-one video coaching sessions with a dietitian and image-based dietary self-monitoring with text message feedback improved overall variety within core food group intake and a reduction in total energy intake from non-core energy-dense, nutrient-poor foods compared to lower level personalization where individuals were self-directed to make dietary changes. In addition, within-group changes were higher for those in the HiP group with change in 12 dietary variables being statistically significant compared to one variable in the LoP group.

In addition to the AES personalized dietary feedback report, the HiP group used a more intensive mode of personalization through a combination of one-on-one coaching by a dietitian via video call and dietary self-monitoring via an image-based food record. Recent reviews of dietitian-led telehealth interventions in adults with chronic diseases have demonstrated the efficacy of this delivery mode for improving dietary patterns or whole food intakes, along with clinical markers such as anthropometry and blood pressure [27]. Our findings provide further evidence that telehealth delivered by a dietitian is effective at improving the dietary intakes of adults. Further, the use of a structured, briefer format consult, in addition to the PNQ and associated toolbox, are unique aspects of this current study. For Australian dietitians in private practice, the average duration for initial and review consultations is 52 and 28 min, respectively [28]. Delivering traditional consultation formats in a briefer consultation time has been recognized as a challenge by dietitians in private practice [29]. We have proposed a framework for a nutrition care model that leverages the advantages of telehealth and other web and mobile technologies [30]. Further, dietitians consider technology important to their practice, acknowledging benefits relating to administrative and clinical tasks [31], however a lack of experience, cost and time are seen as barriers to maximizing use [32].

The comparatively shorter consultation durations used in the current study tested a potential solution to address challenges experienced by Australian dietitians related to consultations. Data collected in electronic form prior to the initial video call session included demographic information, current weight status, physical activity, cooking and shopping behaviors, and barriers to healthy eating via the PNQ, in addition to dietary intake via the AES FFQ. Mobile and web technologies should be seen as tools to complement, rather than compete with, current dietetic practice [30]. By collecting data prior to the consult, the dietitian can spend more of the one-on-one time delivering personalized behavior counselling and nutrition education strategies. The implications of the increasing ubiquity of information and communication technologies in daily life provide an opportunity for dietitians to focus more on higher level skills (e.g., counselling) less impacted by automation [33]. The process evaluation results for the video calls demonstrate that participants were satisfied with the level of personalization and felt these sessions were useful. Of interest, after the initial session, the longer week six session (30 min duration) had a higher rate of attendance compared to the week three session (15 min session), while no participants opted-in for the week nine session. This may suggest individuals prefer more contact at the start of an intervention.

In general, the dietary self-monitoring component consisting of a smartphone image-based food record that was utilized by the HiP group was positively evaluated by participants. Tools for dietary self-monitoring, where feedback on collected intake data is required in order for users to modify their intake, that use image-based food records are in their infancy compared to written food diaries or logging foods in a database as part of a smartphone app. A review of consumer apps that utilized image-based food records for self-monitoring concluded that few apps contained features that supported dietary behavior change [34]. The approach in the current study differed, as food records were reviewed once per week by the research team using a dedicated tool and process, with text message feedback personalized to participants accordingly. Participant feedback on the text messages indicate that a greater variety in messages is needed and may promote improved engagement. Further, manual review of the self-monitoring records has limitations for implementation at scale; however, deep learning applications continue to progress automation of the analysis of image-based and image-assisted dietary assessment methods [35].

For the LoP group, the AES dietary feedback report as a tool to support self-directed changes to diet does not appear to support individuals to change the variety of core, healthful foods consumed as measured by the ARFS. While positive within group changes in the proportion of energy intake derived from core and non-core foods were noted, only the change in intake of baked products was significant (−3.2% (−5.4% to 1.1%; *p* < 0.01) for those in the LoP group. It is unclear why participants in the self-directed group did not address variety within core food groups in their diets; however, responses to the process evaluation may offer some insights. Between-group differences in the process evaluation of the AES feedback report were most notable in the perceived usefulness of the report. Compared to the LoP group, almost twice as many participants in the HiP group either agreed or strongly agreed that the AES contained sufficient information to support making changes to their diet (47.6% vs. 81.8%, respectively). Whilst two-thirds of LoP group participants would have preferred for the AES report to contain more personalized dietary recommendations, only about one-quarter of the HiP group felt this way. The higher levels of agreement with statements on the usefulness of the AES report for the HiP group is likely related to the explanation of the report provided to participants by the dietitian during the video call. In addition, the dietitian assisted the participants to set goals aligned with self-identified priority areas and provided personalized advice and strategies to address their barriers.

Participants in both the LoP and HiP groups reported seeking additional support from external resources such as websites and apps. Some individuals in the HiP group were referred to specific external online resources by the dietitian and it is possible that these may have been reported as additional resources when responding to this question in the process evaluation. However, seeking further information online to clarify information provided by a health professional is not uncommon. For example, one study reported 55% of Australian adults with a chronic disease sought additional information following a consultation with a health professional [36]. It is possible that participants in the current study also sought additional online information for this purpose. Despite this, our results should be interpreted in the context that additional external resources were used by some participants in both groups. Further, this finding raises an interesting point on the potential for interference from external digital tools and/or health professionals in the delivery of digital health interventions and, in turn, the possibility that intervention fidelity may be impacted.

Similar to the AES feedback report, the Food4Me study developed a system to generate personalized feedback from dietary intake data collected via the web [37]. However, the Food4Me system also contained other features to automate the selection of goals and populate some text sections of the report. Evaluation of the automated system in comparison to the same feedback generated manually by the Food4Me research team demonstrated high agreement and feasibility of an automated dietary feedback system. eNutri is another automated web app that uses diet quality scores calculated from a FFQ to personalize nutrition advice [38]. In terms of the COM-B model [15], both Food4Me and eNutri appear more focused on capability aspects by providing information to address knowledge, as opposed to motivational and environmental factors. The PNQ and toolbox attempt to address these issues by incorporating a more complete model of behavior change, which is personalized at the individual level. The PNQ and toolbox were used as tools to support the dietitian in the delivery of brief coaching sessions, and has since been adapted for use in other population groups [39,40]. Potential exists for the PNQ and associated toolbox to be integrated into an online self-administered dietary assessment platform to automate delivery of more personalized nutrition support at a larger scale. Further, if used as a self-administered resource, the PNQ and intervention toolbox could provide an initial low resource contact point, which has potential for high reach with potential to triage the provision of one-on-one support by a dietitian.

There are some limitations in the current study that should be acknowledged. For most participants, the highest qualification obtained was at the postgraduate level, which reflects the university setting and the associated participation of staff and students. As a result, this limits the generalizability of the findings to the wider population. As a self-administered measure, the AES FFQ is subject to inherent errors of self-reported dietary data [41]. Further, there is some evidence that participation in an intervention can influence reporting of dietary intake [42] and findings from this study should be interpreted in this context. 

## 5. Conclusions

The current study has demonstrated that personalized nutrition support delivered using technology can improve dietary intake in adults with significantly greater changes observed in dietary intakes for those receiving a higher level of personalization. A higher level of personalized support, which included the AES dietary feedback report, and one-on-one dietitian video calls with self-monitoring, resulted in greater dietary change compared to the AES report alone. These findings suggest dietary technologies in combination with dietitian delivered advice have a greater impact compared to when used in a self-directed format. Further research should be conducted in more diverse population groups. 

## Figures and Tables

**Table 1:** Participant (*n* = 50) baseline characteristics.

Variables	Mean (SD) or Count (%)	
	HiP Group (*n* = 26)	LoP Group (*n* = 24)
Sex		
Female	23 (88.5%)	20 (83.3%)
Male	3 (11.5%)	4 (16.7%)
Age (years)	37.3 (11.6)	41.3 (13.2)
Height (m)	1.7 (0.1)	1.7 (0.1)
Weight (kg)	71.8 (15.9)	75.2 (18.5)
BMI (kg/m^2^)	25.6 (5.0)	27.3 (6.9)
Born in Australia	20 (76.9%)	18 (75.0%)
Highest Level of Education Obtained		
High School Certificate (Year 10/ Year 12)	3 (11.5%)	2 (8.3%)
Diploma or Certificate	4 (15.4%)	3 (12.5%)
Bachelors degree	7 (26.9%)	6 (25.0%)
Postgraduate degree	12 (46.2%)	13 (54.2%)
Employment status		
Full-time	8 (30.8%)	13 (54.2%)
Part-time	9 (34.6%)	6 (25.0%)
Casual or other type of work	5 (19.2%)	2 (8.3%)
Not currently in paid employment	1 (3.8%)	2 (8.3%)
Student	3 (11.5%)	1 (4.2%)
Income (Individual Level)		
≤$41,599/year	11 (42.3%)	6 (25.0%)
$41,600–$77,999/year	8 (30.8%)	5 (20.8%)
≥$78,000/year	6 (23.1%)	10 (41.7%)
Not reported	1 (3.8%)	3 (12.5%)
Martial Status		
Married or living with a partner	16 (61.5%)	15 (62.5%)
Divorced	2 (7.7%)	2 (8.3%)
Never married	8 (30.8%)	7 (29.2%)
eHEALS [26] (out of 40)	29.8 (5.1)	29.8 (4.9)

**Table 2:** Participant (*n* = 50) baseline dietary intake.

Variables	Mean (SD)	
	HiP Group (*n* = 26)	LoP Group (*n* = 24)
ARFS: overall (out of 73)	36.0 (10.0)	36.8 (9.1)
ARFS: vegetable sub-scale (out of 21)	14.6 (4.3)	14.7 (4.7)
ARFS: fruit sub-scale (out of 12)	5.9 (2.9)	5.9 (3.0)
ARFS: Meat, fish, poultry and other flesh foods (out of 7)	2.7 (1.3)	2.7 (1.5)
ARFS: vegetarian protein sources (out of 6)	2.4 (1.8)	2.8 (1.0)
ARFS: grains, breads and cereals (out of 13)	5.2 (2.0)	5.4 (2.1)
ARFS: dairy (out of 11)	3.8 (2.1)	3.9 (1.7)
ARFS: sauces and spreads (out of 2)	0.8 (0.7)	0.6 (0.7)
ARFS: water (out of 1)	0.6 (0.5)	0.8 (0.4)
Proportion of energy intake from Core Foods (%)	64.7 (11.8)	69.1 (13.5)
Proportion of energy intake from Non-Core Foods (%)	35.3 (11.8)	30.9 (13.5)
Proportion of energy intake from protein (%)	19.4 (4.0)	19.0 (3.0)
Proportion of energy intake from carbohydrates (%)	43.5 (7.3)	41.7 (6.1)
Proportion of energy intake from fats (%)	34.9 (5.2)	35.7 (5.1)
Proportion of energy intake from saturated fats (%)	14.3 (3.3)	14.1 (3.7)
Proportion of energy intake from alcohol (%)	2.3 (3.9)	3.6 (5.7)

**Table 3:** Change in dietary intake.

Variables	HiP Group within-Group Difference		LoP Group within-Group Difference		Between-Group Difference		Group × Time
	Mean (95%CI)	*p*-Value	Mean (95%CI)	*p*-Value	Mean (95% CI)	Sig	*p*-Value
ARFS: overall (out of 73)	5.0 (2.1 to 8)	0.001	−0.6 (−3.7 to 2.6)	0.71	5.6 (1.3 to 10)	0.01	0.01
ARFS: vegetable sub-scale (out of 21)	1.5 (0.1 to 3)	0.04	−0.2 (−1.7 to 1.4)	0.82	1.7 (−0.4 to 3.9)	0.11	0.11
ARFS: fruit sub-scale (out of 12)	0.5 (−0.4 to 1.5)	0.28	0.8 (−0.2 to 1.9)	0.12	−0.3 (−1.7 to 1.2)	0.69	0.69
ARFS: Meat, fish, poultry and other flesh foods (out of 7) ^1^	0.7 (0.2 to 1.2)	0.004	−0.2 (−0.7 to 0.3)	0.34	0.9 (0.2 to 1.6)	0.01	0.01
ARFS: vegetarian protein sources (out of 6)	0.4 (0 to 0.8)	0.06	−0.4 (−0.8 to 0.1)	0.13	0.8 (0.1 to 1.4)	0.021	0.02
ARFS: grains, breads and cereals (out of 13) ^1^	0.8 (−0.1 to 1.7)	0.10	−0.5 (−1.4 to 0.5)	0.31	1.3 (−0.1 to 2.6)	0.06	0.06
ARFS: dairy (out of 11)	1.2 (0.5 to 1.9)	0.001	−0.1 (−0.8 to 0.6)	0.78	1.3 (0.3 to 2.3)	0.01	0.01
ARFS: sauces and spreads (out of 2)	−0.2 (−0.5 to 0.1)	0.12	−0.2 (−0.5 to 0.1)	0.13	0.0 (−0.4 to 0.4)	0.99	0.99
ARFS: water (out of 1) ^1^	0.8 (−0.1 to 0.2)	0.26	−0.1 (−0.2 to 0.1)	0.45	0.1 (−0.1 to 0.3)	0.19	0.19
Proportion of energy intake from Core Foods (%) ^1^	11.7 (7.2 to 16.2)	0.001	4.5 (−0.3 to 9.3)	0.07	7.2 (0.5 to 13.8)	0.04	0.03
Proportion of energy intake from Non-Core Foods (%) ^1^	−11.7 (−16.2 to −7.2)	0.001	−4.5 (−9.3 to 0.3)	0.65	−7.2 (−13.8 to −0.5)	0.04	0.03
Proportion of energy intake from protein (%) ^1^	1.7 (0.7 to 2.8)	0.001	0.9 (−0.2 to 2)	0.10	0.8 (−0.7 to 2.3)	0.30	0.30
Proportion of energy intake from carbohydrates (%) ^1^	−1.6 (−3.8 to 0.6)	0.16	0.1 (−2.3 to 2.4)	0.94	−1.7 (−4.9 to 1.6)	0.31	0.31
Proportion of energy intake from fats (%)	−0.5 (−2.3 to 1.2)	0.56	−0.8 (−2.7 to 1)	0.38	0.3 (−2.3 to 2.9)	0.81	0.81
Proportion of energy intake from saturated fats (%)	−0.8 (−1.7 to 0.2)	0.11	−0.9 (−1.9 to 0.1)	.08	0.1 (−1.3 to 1.5)	0.85	0.85
Proportion of energy intake from alcohol (%) ^1^	−0.2 (−0.8 to 0.4)	0.55	−0.6 (−1.3 to 0)	.06	0.4 (−0.5 to 1.3)	0.32	0.32

^1^ Adjusted within- and between-group differences presented; within-group difference = 3 months–baseline; between-group difference = high personalization (HiP) group–low personalization (LoP) group.

## Supplementary Materials

The following are available online at https://www.mdpi.com/2072-6643/12/11/3334/s1, Table S1. Baseline dietary intake, Table S2. Change in dietary intake, Table S3. Participant (*n* = 22) perceptions of dietitian video coaching sessions, Table S4. Participant (*n* = 22) perceptions of image-based dietary self-monitoring, Table S5. Participant (*n* = 22) perceptions of text message feedback.
