# Supplementary material for: Impact on Dietary Intake of Two Levels of Technology-Assisted Personalized Nutrition: A Randomized Trial
Source: Nutrients. 2020 Oct 29;12(11):3334. doi: 10.3390/nu12113334 (PMC7693517; doi:10.3390/nu12113334)
Supplement: Supplementary file 1 [file nutrients-12-03334-s001.pdf]

Supplementary Materials

Supplementary Table S1. Baseline dietary intake.

	**HiP group (n=26)**	**LoP group (n=24)**
Fruit (serves/day)	2.3 (1.8)	2 (1.1)
Vegetables (serves/day)	4.5 (1.9)	4.7 (1.9)
Energy Intake (kJ/day)	8220 (2721)	8111 (2790)
Proportion of energy intake from vegetables (%)	9.7 (3.5)	10.6 (5.2)
Proportion of energy intake from fruits (%)	9.3 (5.2)	9.1 (5.6)
Proportion of energy intake from meat/flesh foods (%)	13.9 (9.0)	12.9 (7.4)
Proportion of energy intake from vegetarian protein foods (%)	6.4 (4.7)	7.0 (4.6)
Proportion of energy intake from grains, breads and cereals (%)	15 (8.1)	17.1 (6.0)
Proportion of energy intake from dairy foods (%)	10.3 (6.2)	12.6 (8.5)
Proportion of energy intake from sweetened drinks (%)	1.5 (2.6)	0.4 (.8)
Proportion of energy intake from packaged snacks (%)	1.7 (1.4)	2.2 (3.0)
Proportion of energy intake from confectionary (%)	6.4 (8.6)	5.6 (6.0)
Proportion of energy intake from baked products (%)	7 (5.4)	5 (4.4)
Proportion of energy intake from takeaway foods (%)	9.3 (6.9)	6.9 (5.0)
Proportion of energy intake from processed and fatty meats (%)	1.2 (1.3)	1.2 (1.1)
Proportion of energy intake from protein (%)	19.4 (4.0)	19 (3.0)
Proportion of energy intake from carbohydrates (%)	43.5 (7.3)	41.7 (6.1)
Proportion of energy intake from fats (%)	34.9 (5.2)	35.7 (5.1)
Proportion of energy intake from saturated fats (%)	14.3 (3.3)	14.1 (3.7)
Proportion of energy intake from alcohol (%)	2.3 (3.9)	3.6 (5.7)

Supplementary Table S2. Change in dietary intake.

	HiP group within-group difference		LoP group within-group difference		Between-group difference		Group x Time
	Mean (95%CI)	P value	Mean (95%CI)	P value	Mean (95% CI)	Sig	P value
Fruit (serves/day)	0.1 (-0.3 to 0.5)	.55	0.2 (-0.2 to 0.6)	.38	-0.1 (-0.7 to 0.5)	.82	.82
Vegetables (serves/day)	0.5 (-0.3 to 1.4)	.22	0.6 (-0.3 to 1.5)	.21	0.0 (-1.3 to 1.2)	.95	.95
Energy Intake (kJ/day) [1]	-489 (-1142 to 164)	.14	-538 (-1234 to 157)	.13	49 (-919.2 to 1018)	.92	.92
Proportion of energy intake from vegetables (%)[1]	1.4 (-0.3 to 3)	.11	1.6 (-0.2 to 3.4)	.07	-0.2 (-2.7 to 2.2)	.84	.84
Proportion of energy intake from fruits (%)	1.9 (0.2 to 3.5)	.03	1.4 (-0.3 to 3.2)	.11	0.4 (-2.1 to 2.9)	.74	.74
Proportion of energy intake from meat/flesh foods (%)[1]	1.8 (-0.6 to 4.1)	.15	0.9 (-1.7 to 3.4)	.49	0.9 (-2.7 to 4.4)	.63	.63
Proportion of energy intake from vegetarian protein foods (%)	1.3 (-0.1 to 2.7)	.07	1.1 (-0.4 to 2.6)	.16	0.2 (-1.9 to 2.3)	.84	.84
Proportion of energy intake from grains, breads and cereals (%)[1]	2.5 (-0.3 to 5.2)	.08	0.3 (-2.6 to 3.2)	.85	2.2 (-1.8 to 6.3)	.28	.28
Proportion of energy intake from dairy foods (%)	3.0 (0.2 to 5.7)	.04	-0.8 (-3.7 to 2.1)	.59	3.8 (-0.3 to 7.9)	.07	.07
Proportion of energy intake from sweetened drinks (%)	-1.0 (-1.8 to -0.3)	.006	0.0 (-0.7 to 8.2)	.92	-1.1 (-2.2 to 0)	.05	.05
Proportion of energy intake from packaged snacks (%)	-0.1 (-1 to 0.8)	.76	-0.7 (-1.6 to 0.3)	.16	0.5 (-0.8 to 1.9)	.42	.42
Proportion of energy intake from confectionary (%)[1]	-2.3 (-4.8 to 0.2)	.07	-1.2 (-3.9 to 1.4)	.36	-1.1 (-4.8 to 2.6)	.56	.56
Proportion of energy intake from baked products (%)[1]	-5.1 (-7.3 to -3)	<.001	-3.2 (-5.4 to -1.1)	.003	-1.9 (-4.3 to 0.5)	.12	.12
Proportion of energy intake from takeaway foods (%)	-3 (-5 to -0.9)	.006	0.5 (-1.8 to 2.7)	.69	-3.4 (-6.5 to -0.3)	.03	.03
Proportion of energy intake from processed and fatty meats (%)[1]	0.0 (-0.7 to 0.7)	>.99	0.3 (-0.5 to 1.1)	.46	-0.3 (-1.4 to 0.8)	.59	.59

[1] Adjusted within- and between-group differences presented; within-group difference = 3 months – Baseline; Between-group difference = HiP group – Lo P group.

Supplementary Table S3. Participant (n=22) perceptions of dietitian video coaching sessions.

	Count (%)				
**Statements (as asked)**	**Strongly disagree**	**Disagree**	**Neither disagree or agree**	**Agree**	**Strongly agree**
The video coaching sessions with the dietitian, provided me with useful information about nutrition	0 (0.0%)	0 (0.0%)	1 (4.5%)	12 (54.5%)	9 (40.9%)
The video coaching sessions with the dietitian, increased my confidence to improve my diet and eating behaviors	0 (0.0%)	0 (0.0%)	5 (22.7%)	9 (40.9%)	8 (36.4%)
The video coaching sessions with the dietitian, helped me to achieve my goals	0 (0.0%)	2 (9.1%)	3 (13.6%)	11 (50.0%)	6 (27.3%)
The video coaching sessions with the dietitian, motivated me to eat better	0 (0.0%)	1 (4.5%)	4 (18.2%)	9 (40.9%)	8 (36.4%)
The video coaching sessions with the dietitian, made me feel accountable	1 (4.5%)	1 (4.5%)	2 (9.1%)	13 (59.1%)	5 (22.7%)
I felt that my goals were personalized to my needs	0 (0.0%)	1 (4.5%)	2 (9.1%)	14 (63.6%)	5 (22.7%)
I felt that the strategies suggested by the dietitian addressed the barriers that were preventing me from eating healthily	0 (0.0%)	1 (4.5%)	5 (22.7%)	11 (50.0%)	5 (22.7%)
The email summary of my goals and strategies that I received after the coaching sessions were useful	0 (0.0%)	1 (4.5%)	1 (4.5%)	16 (72.7%)	4 (18.2%)
I would use video-consultations again to consult a dietitian	0 (0.0%)	4 (18.2%)	2 (9.1%)	12 (54.5%)	4 (18.2%)
I would recommend to others to consult a dietitian via video calls.	0 (0.0%)	2 (9.1%)	4 (18.2%)	13 (59.1%)	3 (13.6%)
I found the video coaching sessions with the dietitian useful	0 (0.0%)	2 (9.1%)	1 (4.5%)	14 (63.6%)	5 (22.7%)
Delivering the coaching sessions via video call made it easier to access the dietitian than if attending the session in-person.	0 (0.0%)	1 (4.5%)	2 (9.1%)	10 (45.5%)	9 (40.9%)

Supplementary Table S4. Participant (n=22) perceptions of image-based dietary self-monitoring.

	Count (%)				
**Statements (as asked)**	**Strongly disagree**	**Disagree**	**Neither disagree or agree**	**Agree**	**Strongly agree**
Using the Evernote app to take images of my food and drinks was easy	1 (4.5%)	4 (18.2%)	1 (4.5%)	15 (68.2%)	1 (4.5%)
Using the Evernote app, to add a description of the foods and drinks in the image was easy	1 (4.5%)	5 (22.7%)	3 (13.6%)	11 (50.0%)	2 (9.1%)
On the days I recorded my diet, remembering to take an image of my meals before eating was easy	2 (9.1%)	8 (36.4%)	4 (18.2%)	8 (36.4%)	0 (0.0%)
On the days I recorded my diet, remembering to take an image of my snacks before eating was easy	3 (13.6%)	8 (36.4%)	5 (22.7%)	6 (27.3%)	0 (0.0%)
On the days I recorded my diet, remembering to take an image of any leftover food or drink was easy	2 (9.1%)	9 (40.9%)	4 (18.2%)	7 (31.8%)	0 (0.0%)
I found the marker (the card with the colored squares) easy to carry around	0 (0.0%)	1 (4.5%)	3 (13.6%)	12 (54.5%)	6 (27.3%)
I found it easy to remember to include the marker (the card with the colored squares) in image with my meals and snacks	0 (0.0%)	2 (9.1%)	3 (13.6%)	14 (63.6%)	3 (13.6%)
The weekly text messages reminding me to self-monitor my diet were helpful	0 (0.0%)	4 (18.2%)	8 (36.4%)	5 (22.7%)	5 (22.7%)
Self-monitoring my diet using the image-based food record did not interfere with my daily activities	2 (9.1%)	8 (36.4%)	4 (18.2%)	8 (36.4%)	0 (0.0%)
Self-monitoring my diet using the image-based food record did not interfere with my social interactions	1 (4.5%)	9 (40.9%)	6 (27.3%)	5 (22.7%)	1 (4.5%)
Self-monitoring my diet using the image-based food record increased my awareness of the types of foods I was eating	0 (0.0%)	1 (4.5%)	3 (13.6%)	10 (45.5%)	8 (36.4%)
Self-monitoring my diet using the image-based food record increased my awareness of how often I was eating	0 (0.0%)	1 (4.5%)	3 (13.6%)	12 (54.5%)	6 (27.3%)
Self-monitoring my diet using the image-based food record increased my awareness of the amounts of foods I was eating	0 (0.0%)	3 (13.6%)	2 (9.1%)	11 (50.0%)	6 (27.3%)
Self-monitoring my diet using the image-based food record assisted me with achieving my healthy eating goals	0 (0.0%)	4 (18.2%)	7 (31.8%)	8 (36.4%)	3 (13.6%)
Self-monitoring my diet using the image-based food record was acceptable	1 (4.5%)	4 (18.2%)	4 (18.2%)	10 (45.5%)	3 (13.6%)

Supplementary Table S5. Participant (n=22) perceptions of text message feedback.

	Count (%)				
**Statements (as asked)**	**Strongly disagree**	**Disagree**	**Neither disagree or agree**	**Agree**	**Strongly agree**
The weekly text message feedback made me think about the variety of vegetables I eat	0 (0.0%)	0 (0.0%)	1 (4.5%)	13 (59.1%)	8 (36.4%)
The weekly text message feedback made me think about the amount of vegetables I eat	0 (0.0%)	0 (0.0%)	0 (0.0%)	13 (59.1%)	9 (40.9%)
The weekly text message feedback made me think about the variety of fruits I eat	0 (0.0%)	2 (9.1%)	1 (4.5%)	12 (54.5%)	7 (31.8%)
The weekly text message feedback made me think about the amounts of fruits I eat	0 (0.0%)	0 (0.0%)	1 (4.5%)	12 (54.5%)	9 (40.9%)
The weekly text message feedback made me think about the amounts of junk foods I eat	1 (4.5%)	2 (9.1%)	1 (4.5%)	9 (40.9%)	9 (40.9%)
The two text messages that asked to reflect on progress towards my goals were useful	0 (0.0%)	7 (31.8%)	2 (9.1%)	8 (36.4%)	5 (22.7%)
The text message feedback assisted me with achieving my healthy eating goals	0 (0.0%)	6 (27.3%)	5 (22.7%)	8 (36.4%)	3 (13.6%)
The text messages on my diet were useful in helping me understand my diet	0 (0.0%)	6 (27.3%)	3 (13.6%)	10 (45.5%)	3 (13.6%)
The text messages on my diet helped to motivate me to change my diet	1 (4.5%)	6 (27.3%)	4 (18.2%)	7 (31.8%)	4 (18.2%)
The text messages made me think about the types of foods I eat but only for a short while	0 (0.0%)	4 (18.2%)	5 (22.7%)	10 (45.5%)	3 (13.6%)
The text messages on my diet made me feel better about my diet	0 (0.0%)	6 (27.3%)	5 (22.7%)	9 (40.9%)	2 (9.1%)
The text messages on my diet made me feel worse about my diet	3 (13.6%)	7 (31.8%)	5 (22.7%)	7 (31.8%)	0 (0.0%)
